# Increased Frequency of Spontaneous Neoplastic Transformation in Progeny of Bystander Cells from Cultures Exposed to Densely Ionizing Radiation

**DOI:** 10.1371/journal.pone.0021540

**Published:** 2011-06-28

**Authors:** Manuela Buonanno, Sonia M. de Toledo, Edouard I. Azzam

**Affiliations:** 1 Department of Radiology, University of Medicine and Dentistry of New Jersey (UMDNJ), New Jersey Medical School Cancer Center, Newark, New Jersey, United States of America; 2 University of Medicine and Dentistry of New Jersey (UMDNJ), Graduate School of Biomedical Sciences, Newark, New Jersey, United States of America; University of Medicine and Dentistry of New Jersey, United States of America

## Abstract

An increased risk of carcinogenesis caused by exposure to space radiation during prolonged space travel is a limiting factor for human space exploration. Typically, astronauts are exposed to low fluences of ionizing particles that target only a few cells in a tissue at any one time. The propagation of stressful effects from irradiated to neighboring bystander cells and their transmission to progeny cells would be of importance in estimates of the health risks of exposure to space radiation. With relevance to the risk of carcinogenesis, we investigated, in model C3H 10T½ mouse embryo fibroblasts (MEFs), modulation of the spontaneous frequency of neoplastic transformation in the progeny of bystander MEFs that had been in co-culture 10 population doublings earlier with MEFs exposed to moderate doses of densely ionizing iron ions (1 GeV/nucleon) or sparsely ionizing protons (1 GeV). An increase (*P*<0.05) in neoplastic transformation frequency, likely mediated by intercellular communication through gap junctions, was observed in the progeny of bystander cells that had been in co-culture with cells irradiated with iron ions, but not with protons.

## Introduction

The experience gained from space missions over the past 50 years has highlighted the necessity of humans for the exploration of deep space [1]. However, the health risks, in particular the increased risk of cancer, that may be associated with exposure to space radiation represents a major limitation to this activity [2]. To control and mitigate these risks, the National Aeronautics and Space Agency (NASA) has emphasized the need for ground-based characterization of the biological effects of space radiation and elucidation of the mediating mechanisms. These studies must be investigated in the context of low dose, low fluence, and low dose-rate effects.

During interplanetary missions, astronauts are exposed to different types of radiation [3]. Galactic cosmic rays ranging from energetic protons of low linear energy transfer (LET), to helium particles and particles of high charge and high energy (HZE) with high LET, as well as transient radiation from solar particle events (protons and small percentage of energetic ions) are the primary source of ionizing radiation in outer space.

Though less abundant than sparsely ionizing protons, HZE particles are highly ionizing with much greater potential to induce cell and tissue injury. The chemical reactants that they produce at high concentration along their trajectory interact efficiently with cellular molecules causing both covalent modifications and changes in cell signaling [4]. The net effect is clustered damage in DNA and other molecules [5] that occur shortly after irradiation and can extend along a long column of cells in tissue [6], and perturbation of oxidative metabolism that results in delayed damage [7]. Clustered DNA damage challenges repair systems and often is misrepaired (reviewed in [8]) leading to chromosomal changes that may predispose to cancer. Therefore, the risk of carcinogenesis from exposure to low fluence HZE particles may be greater in magnitude than caused by low doses of common terrestrial forms of radiation, such as γ rays [9], [10]. Epidemiological studies in humans would be ideal to evaluate the health risks induced by exposure to space radiation. However, such studies are likely to remain limited for years to come, given the relatively insignificant number of humans exposed to HZE particles. Thus, experimental studies in animal and cell culture models are essential to understand biological effects and their underlying mechanisms, which may help estimate corresponding risks for humans [11]. Such investigations would complement modeling studies based on extrapolation of epidemiological data obtained from the survivors of the A-bomb, nuclear accidents, and industrial activities involving exposure to radiation [12].

Risk estimates of carcinogenesis induced by space radiation, particularly at low doses and low fluences, are further complicated by accumulating experimental evidence indicating that the deleterious effects of certain radiation types (e.g. alpha or HZE particles) may manifest also in non-irradiated bystander cells in the exposed population (reviewed in [13]). Important biological responses, including genetic changes, have been shown to arise in non-irradiated cells that were in the vicinity of directly targeted cells or recipient of growth medium from irradiated cell cultures [14]. Recently, stressful bystander effects were shown to be induced by the HZE component of space radiation [15], [16]. Furthermore, such effects are propagated in progeny cells [17]. Progeny of bystander cells from normal human cell cultures exposed to HZE particles (1 GeV/nucleon iron ions or 600 MeV/nucleon silicon ions) harbored higher levels of chromosomal damage and oxidative stress, as judged by protein carbonylation and lipid peroxidation, than respective control cells [17]. DNA damage and oxidative stress act at all stages of carcinogenesis [18]. However, the risk of neoplastic transformation in bystander cells remains unclear; if it exists, it would have profound implications for the health risks associated with human space exploration. It would show that the risk is greater than predicted solely from the biological responses triggered in the targeted cells.

Using a model cell culture system to investigate malignant transformation [19], here we show that 1 GeV/nucleon iron ions induce a significant increase in the frequency of spontaneous neoplastic transformation in the progeny of bystander C3H 10T½ mouse embryo fibroblasts (MEFs),when assayed 10 generations after they had been in co-culture with irradiated C3H 10T½ MEFs.

## Results and Discussion

Using a layered tissue culture system [17], we measured the frequency of spontaneous neoplastic transformation in the progeny of bystander C3H 10T½ MEFs that had been in co-culture 10 population doublings earlier with confluent MEFs that were irradiated with energetic iron ions (1 GeV/nucleon). Briefly, C3H 10T½ cells destined to be bystanders were seeded onto inverted Transwell® inserts with 3 µm pores. Following attachment, the inserts were inverted and placed into the wells of plates and cultured to form an adherent confluent monolayer. Irradiated or sham-treated cells, derived from confluent cultures maintained in flasks, were harvested within 10 min after exposure to a 25 cGy dose from energetic iron ions. At this dose, most cells (87%) in the exposed population were traversed through the nucleus on average by 2 iron ion tracks, which would result in an absorbed dose of ∼30 cGy/cell [20]. The harvested cells were then seeded at confluent density on top of the insert with confluent bystander cells growing at the bottom of it. Within 2 h after plating, irradiated cells adhered and formed functional junctional channels with bystander cells, as assessed by the transfer of calcein dye [21]. Irradiated and bystander cells may also communicate with each other through diffusible factors transferred across the pores of the membrane. The directly irradiated and bystander cells were left in co-culture for a total of 5 h. Subsequently, bystander cells were harvested, grown, and serially subcultured for progeny studies upon reaching the confluent state. Control experiments whereby the irradiated cells were labeled with a cell tracker dye (Cell Tracker Orange CMTMR) confirmed that these cells do not move through the 3 ìm pores of the membrane, and the purity of the bystander population was confirmed by microscopy and flow cytometry analyses. In order to include all the progeny cells derived from the initial bystander cell population in the analyses, at each passage, the cells were subcultured in increasingly larger size flasks upon reaching the confluent state. Following 10 population doublings that occurred in ∼2 weeks, the cells were harvested and assayed for clonogenic survival and neoplastic transformation. When scoring transformed foci, type II or III foci according to the classification of Reznikoff *et al.* [19] were considered in the analyses.

**Figure 1:**
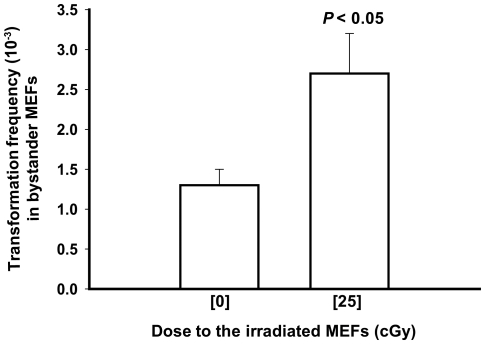
Spontaneous neoplastic transformation frequency in the progeny of bystander C3H 10T½ MEFs. Co-culture of bystander C3H 10T½ MEFs with MEFs exposed to 0 or 25 cGy of 1 GeV/nucleon iron ions results in increased frequency of spontaneous neoplastic transformation in the distant progeny of the bystander cells. Neoplastic transformation frequencies and standard errors of the mean (SEM) were calculated with the null method applied to combined data from 3 independent experiments.

The frequency of neoplastic transformation was evaluated by three different methods, namely transformation frequency per viable cell, per cell at risk, and by applying the null method [22] to the combined results of 3 independent experiments (Table 1). In every case, relative to control, a significant increase in the spontaneous neoplastic transformation frequency was observed. This increase was associated with an increase (*P*<0.01) in cell viability (Table 1, 14.1±0.6% in bystander cells co-cultured with irradiated cells vs. 8.2±1.0% in bystander cell co-cultured with sham-irradiated cells). When assessed by the null method, the progeny of bystander cells expressed a 2-fold increase (within 95% confidence interval) in transformation frequency [(2.7±0.5)×10^−3^ vs. (1.3±0.2) x 10^−3^ in control] (Figure 1). Similarly, the increase in spontaneous neoplastic potential in bystander cells was significant when the data were analyzed in terms of the number of foci per flask (*P*<0.0001) or the number of flasks without foci (*P*<0.005) (Table 2).

**Table 1:** Frequency of spontaneous neoplastic transformation in bystander cells.

	Dose to irradiated cells [cGy]	% survival (± SD)	Flasks	Foci	Flasks without foci	Viable cells	Cells at risk	Transformation frequency (10^−3^)			95% confidence intervals (10^−3^)
								Per viable cells (± SE)	Per cells at risk	Null method(± SEM)	
Exp. 1	**[0]**	6.2 (0.3)	13	7	8	657	10608	10.6 (4.0)	0.7	9.6 (0.2)	
Exp. 2	**[0]**	9.1 (0.3)	16	16	7	1459	16032	11.0 (2.8)	1.0	9.0 (0.3)	
Exp. 3	**[0]**	9.3 (1.0)	14	22	5	1269	13650	17.0 (3.7)	1.7	11.4 (0. 4)	
	**Sum**		**43**	**45**	**20**	**3385**	**40290**	**13.3 (2.0)**	**1.1**	**1.3 (0.2)**	**1.0–1.6**
Exp. 1	[**25**]	15.3 (0.8)	15	30	2	2180	14280	13.8 (2.5)	2.1	13.9 (0.7)	
Exp. 2	[**25**]	13.2 (1.4)	15	94	1	2057	15585	45.7 (4.8)	6.0	19.7 (1.0)	
Exp. 3	[**25**]	13.7 (3.2)	16	123	1	1977	14384	62.2 (5.8)	8.6	22.4 (1.0)	
	**Sum**		**46**	**247**	**4**	**6214**	**44250**	**39.7 (1.8)**	**5.6**	**2.7 (0.5)**	**1.7–3.6**

Neoplastic transformation frequency in the progeny of bystander C3H 10T½ MEFs assayed 10 population doublings after they had been in co-culture with 1 GeV/nucleon iron ion- or sham-irradiated MEFs. Data from each independent experiment are shown separately. For each of the 3 experiments shown and for their sum, the transformation frequency per viable cell (± standard error, SE), per cell at risk, and using the null method (± standard error of the mean, SEM) is indicated. Relative to control, progeny of bystander C3H 10T½ MEFs that had been in co-culture 10 population doublings earlier with MEFs irradiated with 25 cGy from 1 GeV/nucleon iron ions showed an increased frequency of spontaneous neoplastic transformation.

**Table 2:** Neoplastic transformation in bystander cells expressed as number of foci/flask or flasks without foci.

Dose to the irradiated cells [cGy]	Foci/Flasks^a^			Tot.	*P* b	Flasks without foci/Flasks^a^			Tot.	*P* b
**[0]**	7/13	16/16	22/14	45/43	< 0.0001	7/13	7/16	8/15	20/43	0.002
[**25**]	30/15	94/15	123/16	247/46		1/15	2/15	1/16	4/46	

Spontaneous neoplastic transformation in the progeny of bystander C3H 10T½ MEFs that had been in co-culture, 10 population doublings earlier, with MEFs irradiated with 1 GeV/nucleon iron ions or with sham-irradiated MEFs. The data of 3 independent experiments were summed and analyzed in terms of the number of foci per flask or the number of flasks without foci.

a Data from each independent experiment are shown separately.

b *P* values are calculated with a Fisher's exact test based on comparing the total number of events per flask, when bystander cells were co-cultured with irradiated cells, with the corresponding data from respective control.

Traditionally, the important biological effects of radiation were assumed to arise in directly irradiated cells; bystander cells in the vicinity of irradiated cells would be unaffected [23]. Using an endpoint that is relevant to the risk of carcinogenesis, we show that the stressful effects of energetic iron ions are greater than predicted by traditional assumptions. The above results are opposite to the observations that exposure to low doses of γ rays, a radiation with low LET character, induces protective effects that reduce the transformation frequency to below the spontaneous level [24], [25]. In this context, during space travel or in the course of radiotherapy with HZE particles, low LET δ rays generated from HZE particle traversals may induce signaling events that trigger protective effects that may reduce the risk of neoplastic transformation [26]. Further, during space travel, δ rays are thought to traverse each cell in a human body about once per day [27]. However, when co-cultured with irradiated cells in the layered tissue culture system used in the present study, bystander cells did not receive δ rays. Investigating the effect of sequential exposure to low LET protons or γ rays followed by high LET HZE particles on the frequency of neoplastic transformation in irradiated and bystander cells would complement these studies and is pertinent to radiation protection and to radiotherapy, as patients receive diagnostic procedures prior to treatment [28].

Consistent with the lack of induction of DNA damage and oxidative stress in progeny of bystander cells from cultures exposed to energetic protons, a low LET radiation [17], co-culture of bystander C3H 10T½ MEFs with MEFs exposed to 100 cGy of 1 GeV protons (LET ∼0.2 keV/µm), did not increase the frequency of spontaneous neoplastic transformation above background level. Rather, the transformation frequency calculated by the null method was slightly lower than control [(6.6±0.4)×10^−3^ vs. (7.3±0.2)×10^−3^ in control]. These results are not likely due to alterations in the cloning efficiency of the bystander cells; the cloning efficiency was similar to that of progeny of bystander cells that were co-cultured with iron ion-irradiated cells. These data expand our previous studies showing that the induction of bystander stressful effects strongly depends on the rate of energy deposition per unit length along the particle trajectory (i.e. LET). Doses ranging from 100 to 400 cGy of low LET protons or γ rays did not result in the propagation of stressful bystander effects as assessed by the endpoints of cloning efficiency, micronucleus formation and markers of protein or lipid oxidation [29], [30].

Several mechanisms have been implicated in the propagation of radiation-induced bystander effects. They include oxidative metabolism, direct and indirect modes of intercellular communication, physical contact and likely other factors [31], [32]. Gap junctions linking contiguous cells were shown, by direct approaches, to mediate the propagation of signaling events that result in DNA damage and oxidative stress from cells exposed to α particles (a high LET radiation) to neighboring bystander cells [33]. Similar to irradiation with iron ions, we have also observed that co-culture of C3H 10T½ MEFs exposed to a mean dose of 80 cGy from 3.2 MeV α particles (LET ∼122 keV/µm) with bystander MEFs resulted in increased (*P*<0.05) frequency of spontaneous neoplastic transformation in the progeny of bystander cells [(3.9±0.4)×10^−3^ vs. (0.4±0.3)×10^−3^ in control, calculated by the null method]. Consistent with a prominent role for junctional communication in mediating the observed effect [34], the frequency of transformed cells was reduced [(3.9±0.4 vs. 2.3±0.4)×10^−3^] when the irradiated and bystander cells were co-cultured in the presence of á-glycyrrhetinic acid (AGA), a gap junction inhibitor.

Although it was shown that cells derived from type II and III C3H 10T½ foci form tumors when injected subcutaneously in syngeneic mice [35], [36], the use of results from tissue culture experiments in human health risk assessment must be done with caution. Nevertheless, neoplastic transformation experiments have been useful to predict radiation-induced carcinogenesis in humans exposed to domestic radon [37], and were consistent with risk estimations for leukemia and breast cancer derived by epidemiological findings [38]. Quantitative oncogenic transformation experiments in normal human cells would be preferred to those in MEFs, however, these are difficult to achieve [39].

In summary, our results demonstrate by a biological endpoint that is of relevance to carcinogenesis that stressful bystander effects are induced by the high LET component of space radiation and persist in progeny cells. They extend those of others which suggested that cellular exposure to low fluence á particles induces neoplastic transformation in bystander cells [40]. They show a 2 to 3 fold-increase in neoplastic transformation in progeny of bystander cells (Table 1). This increase is similar to that induced in C3H 10T½ cells targeted by one or two á particles [41], and greater than that observed in HeLa x skin fibroblast cell line CGL1 directly targeted by 1 GeV/nucleon iron ions [26]. In the latter cells, an increase in the transformation frequency occurred at doses greater than 20 cGy to the exposed cell cultures.

In addition to their relevance in estimates of health risks during extended space missions, the results are also pertinent to radiotherapy, as particle therapy with energetic protons or heavy ions (e.g. carbon ions) is increasingly being used in cancer treatment [42], [43]. Neoplastic transformation of progeny of bystander normal cells surrounding the irradiated tumor may increase the risk of second malignancies after treatment. In this respect, it would be informative to expand the current studies with HZE particles and investigate the effect of higher doses relevant to cancer therapy.

## Materials and Methods

### Cells

Mouse embryo fibroblasts, C3H 10T½ clone 8, obtained from the American Type Tissue Culture Collection, were cultured as previously described [44]. Briefly, cells at passage 8–10 were grown in Dulbecco Minimum Essential Medium (DMEM)/F-12 (Mediatech) supplemented with 10% heat-inactivated FCS (CellGro), 2 mM alanyl-L-glutamine, 100 U/mL penicillin and 100 µg/mL streptomycin (Sigma). Cells were routinely maintained in a 37° C humidified incubator in an atmosphere of 5% CO_2_ in air. Cells destined for irradiation were seeded in tissue culture flasks at a density that allowed them to reach the density-inhibited state within 5 days. They were then fed twice on alternate days, and experiments were initiated 24–48 h after the last feeding. Under these conditions, ∼90% of the cells were in G_0_/G_1_ phase of the cell cycle as determined by [^3^H]-thymidine uptake and/or flow cytometry. Synchronization of the cells in G_0_/G_1_ phase by density-inhibition eliminates complications in interpretation of the results, which arises from changes in cellular radiation sensitivity at different phases of the cell cycle [45]. Under these cell culture conditions, the colony forming efficiency of CH3 10 T½ MEFs was ∼15%.

The irradiated cells were co-cultured with confluent bystander cells (∼90% in G_0_/G_1_) grown onto the inverted Transwell inserts. Under these conditions, the colony forming efficiency of the bystander cells varied between 6 and 10%. The cells appeared healthy; they harbored similar level of micronuclei and proliferated at the same rate as cells grown in flasks (doubling time ∼ 16 h).

### Inhibition of Gap Junction Communication

18-α-glycyrrhetinic acid (AGA) (Sigma), a reversible inhibitor of gap junction communication, was dissolved in 99.5% dimethyl sulfoxide (DMSO) and added to cell cultures at a concentration of 50 µM. At this concentration, AGA was not toxic to C3H 10T½ cells incubated in the presence of the drug for 5 h. Control cell cultures were incubated with the dissolving vehicle (0.25% DMSO).

### Irradiation

Iron ion (1 GeV/nucleon^ 56^Fe^26+^) and proton (1GeV ^1^H^+^) irradiations were conducted at the NASA Space Radiation Laboratory (NSRL) located at the Brookhaven National Laboratory (Upton, NY). Description of the facility and radiation beam information can be found at www.bnl.gov/medical/NASA/LTSF.asp. The culture flasks were positioned perpendicular to the beam so that the irradiating particles impacted first the plastic of the culture vessel, followed by the adherent cells and then the growth medium. Confluent cultures were exposed to 25 cGy (50 cGy/min) from iron ions or to 100 cGy (100 cGy/min) from protons. Whereas protons result in uniform irradiation of the cell population, according to Charlton and Sephton [20], exposure to a mean dose of 25 cGy from iron ions results in ∼87% of nuclei (mean nuclear area of a C3H 10T½ cell being ∼203 µm^2^ [46]) in a confluent population being traversed on average by 2 particles (∼15 cGy/particle hit) and 13% of nuclei being unhit. At the place where the culture flasks were positioned, the LET was estimated to be ∼151 keV/µm for iron ions and 0.2 keV/µm for protons. The flasks were filled to capacity with growth medium that was pH- and temperature-equilibrated, 3 h before the radiation exposure. This ensured that during the irradiation, temperature fluctuations were attenuated and the cells were immersed in medium, which alleviates changes in osmolarity and partial oxygen tension, parameters that greatly affect the cellular response to radiation [47], [48]. Control cells were sham-treated and handled in parallel with the test cultures. The experiments with HZE particles were carried out during different NSRL runs between 2008 and 2010, and dosimetry was performed by the NSRL physics staff.

For irradiation with α particles, cells were exposed at 37 °C in a 5% CO_2_ atmosphere to a 0.0002 Ci ^241^Am-collimated source housed in a helium-filled Plexiglas box at a dose rate of 2 cGy/min as we have previously described [21]. Irradiation was carried out from below with α particles with an average energy of 3.2 MeV (LET ∼122 keV/µm) at contact with the cells.

### Neoplastic Transformation

Progeny of bystander C3H 10T½ cells were assayed for clonogenic survival and neoplastic transformation as described [36]. Briefly, for each treatment, fifteen 75 cm^2^ flasks were seeded with ∼1 × 10^3^ cells per flask. The cells were then fed once a week for the subsequent 6–7 weeks with DMEM/F-12 medium supplemented with 5% heat-inactivated FCS. The cultures were then fixed in 95% ethanol and stained with Giemsa (1∶20 in PBS, pH 7.4). Neoplastic transformation frequencies were estimated using morphological criteria. A transformed colony is a focus of highly polar, multilayered criss-crossed array of densely stained cells classified as type II or type III foci [36]. Transformed foci are dense and discrete and can be easily distinguished from the flat background of control cells or type I foci. Further, they induce tumors when injected subcutaneously in mice [35], [36]. To estimate clonogenic survival, three flasks per treatment were fixed following a 9 day-incubation period. Colonies consisting of 50 or more cells were considered in the analyses [49].

Type II and III foci were considered and the data were analyzed by summing the results of 3 separate experiments; pooled data from any condition were compared to pooled data from control samples. Neoplastic transformation frequencies were calculated per viable cell (i.e. colony forming cell) (± the standard error), per cell at risk, and using the null method. The latter calculates the transformation frequency per viable cell, and eliminates possible errors from satellite colony formation as a consequence of the multiple feedings during the 42–49 days post-irradiation expression period. It compares the average number of transformed foci per flask (λ), with the fraction of flasks without foci (*f = n/N*, where *n* is the number of flasks without foci and *N* is the total number of flasks) and assumes that the potential number of foci follows a Poisson distribution (λ =  -ln *f = *-ln (*n/N*)). The transformation frequency is given by λ, *N−S*. = −, ln(*−*,*n*-*N.)*, *N−S..*, where *S* is the total number of viable cells. This method also allows for calculation of the standard error of the mean (SEM) =  ,−(, 1−*n*.−, 1−*N*)., and the 95% confidence interval (twice the standard error of the mean) [22].

In Table 2, the Fisher exact test was used to calculate the *P* values when neoplatic transformation was evaluated by comparing the number of foci per flask or the number of flasks without foci in bystander cells that were co-cultured with sham-irradiated or irradiated cells, respectively. Different approaches were adopted to analyze the frequency of neoplastic transformation as a significant increase may be observed by one method but not the other.
